# Auditory skills and neural encoding in children with speech sound disorder

**DOI:** 10.1590/2317-1782/e20240173en

**Published:** 2025-11-17

**Authors:** Dara Eliza Rohers, Márcia Keske-Soares, Eliara Pinto Vieira Biaggio

**Affiliations:** 1 Departamento de Fonoaudiologia, Universidade Federal de Santa Maria – UFSM - Santa Maria (RS), Brasil.

**Keywords:** Speech Disorders, Electrophysiology, Auditory Processing, Child, Speech Perception, Articulation Disorders

## Abstract

**Purpose:**

To investigate binaural integration and temporal resolution auditory skills, measure parents’ and/or guardians’ perceptions of their dependents’ auditory behavior, and analyze neural encoding in children with speech sound disorder (SSD).

**Methods:**

The study included 28 children divided into two groups: 13 with SSD (mean age of 7 years) and 15 with typical speech development, matched for age with the study group. Auditory skills of binaural integration and temporal resolution were assessed. Parents and/or guardians completed the Auditory Processing Domains Questionnaire. Neural encoding was analyzed using the frequency following response with a /da/ stimulus, assessing amplitudes, absolute latencies, shifts, and the slope measure. The basic frequency analysis of the frequency following response employed the time-frequency distribution of the spectrogram. Inferential data analysis was conducted.

**Results:**

Statistically significant differences were observed in binaural integration auditory skills. However, no such differences were observed in temporal resolution. Parents and/or guardians reported changes in their dependents’ auditory behavior in both groups. In the analysis of neural encoding, children with SSD showed higher latency in the O component, with a greater A-O shift. There was a positive correlation between the severity of SSD and the latency of the E component. The spectrogram analysis revealed greater neuronal excitation in the group with typical development.

**Conclusion:**

Children diagnosed with SSD show alterations in binaural integration auditory skills and in the neural encoding of speech sounds.

## INTRODUCTION

Speech production involves sensory and motor cortical and subcortical networks in the brain, facilitating the integration of auditory information, the representation of sounds, and consequently, the planning and execution of motor acts for sound emission^(1,2)^. Therefore, auditory sensitivity alone is not sufficient for the development of speech perception and production, as the auditory information received about complex sounds must be effectively interpreted^(3)^.

Understanding how the central auditory nervous system (CANS) processes and utilizes the information it receives is fundamental for comprehending how speech disorders manifest in children with speech sound disorders (SSD), especially in phonological disorders (PD)^(4,5)^.

Notably, auditory skills (e.g., binaural integration and temporal resolution) play an important role in the CAP and, by extension, speech. In binaural integration, auditory information received is transmitted to the cerebral hemispheres via the corpus callosum, enabling the interpretation of these acoustic signals. This process facilitates the identification of subtle differences in sounds, as well as the distinction of overlapping sounds in noisy environments, such as speech amidst noise. It should also be mentioned that this highly myelinated structure contains fibers from all sensory modalities and is involved in attention modulation^(6)^.

Conversely, temporal resolution refers to the ability to perceive rapid changes in duration and/or interruptions in auditory stimuli. This skill also encompasses the perception of differences between sounds produced at the same articulatory point, distinguishable only by voicing. It is essential to emphasize that temporal resolution is crucial for the recognition of speech sounds, the perception of variations in duration, pauses, and the speed of syllables^(7)^.

Regarding the neurophysiological evaluation of the auditory pathway, Auditory Evoked Potentials with speech stimuli provide a more robust analysis of the CANS, particularly in assessing the neural encoding of verbal sounds associated with auditory skills and, consequently, communication. In this context, the frequency-following response (FFR) reflects the functioning of cortical and subcortical regions related to speech production^(8,9)^.

Analyzing specific aspects of speech production reveals that phonological acquisition in the language development process is a gradual process that occurs as the child grows, culminating in the appropriate production of all speech sounds. In Brazilian Portuguese, the phonological inventory is complete around the age of five years^(10)^.

Nevertheless, some children may have verbal comprehension difficulties when speech development does not occur as expected or follows an atypical pattern, which may lead to different SSDs. These disorders can include a variety of difficulties or combinations involving perception, motor production, and/or phonological representation of speech segments and their prosodic aspects^(11-15)^.

Phonological disorders, the most prevalent of SSDs in speech-language pathology practice, are characterized by the child’s difficulty in correctly pronouncing words, mainly presenting omissions and/or substitutions of consonants and consonant clusters^(16,17)^, which results in unintelligible speech. Hence, understanding the relationship between auditory and linguistic skills has been a challenge for many researchers, with evidence suggesting the necessity of evaluating information processing in the central auditory pathways beyond purely speech issues in children with PDs^(2,4,17,18)^.

Therefore, investigating the auditory skills related to speech production and their relationship with neural encoding is crucial to contribute to the design of therapeutic processes that emphasize not only speech issues but also the auditory challenges faced by children with PD. As the understanding of the role of auditory information in speech sound production and how auditory perception interacts with motor and sensory systems improves^(2)^, the planning of speech-language therapy strategies for children with PD can be enhanced.

Given the above, this study aimed to analyze binaural integration and temporal resolution skills, measure parents’ and/or caregivers’ perception of their dependents’ auditory behavior, and investigate neural encoding in children with PDs.

## METHODS

This cross-sectional, analytical, and quantitative research was approved by the Research Ethics Committee of a public higher education institution (CAAE no. 68074623.0.0000.5346). The study adhered to all regulatory standards and guidelines for research established by the Brazilian National Health Council. The sample was divided into two groups: the experimental group (EG), consisting of children diagnosed with PDs, and the control group (CG), consisting of children with typical speech acquisition/development.

The speech diagnosis for the children in the EG was conducted beforehand by a qualified team, based on evaluations of lexical-semantic skills, long-term memory in expressive language, receptive vocabulary, phonological assessment using the INFONO, speech motor skills, and Orofacial Myofunctional Evaluation with Scores. Based on the data obtained from the phonological assessment, particularly at the spontaneous naming stage, the severity of the PD was determined by the Percentage of Consonants Correct-Revised (PCC-R), which is classified into four levels: mild (PCC > 85%), mild-moderate (PCC = 65-85%), moderate-severe (PCC = 50-65%), and severe (PCC < 50%)^(19,20)^. The EG comprised children on a waiting list to begin rehabilitation at a speech-language pathology service.

Children for the CG were conveniently recruited using the snowball sampling technique. The children in the CG did not undergo all the evaluations previously mentioned. During interviews with their parents, we confirmed that the children had never received speech therapy, and the family reported no concerns or recognized any delays in their child’s speech development compared to peers. Furthermore, during non-instrumental observations, the responsible researcher did not detect any atypical patterns in the oral production of the evaluated children.

The inclusion criteria for both groups stipulated that the children be aged between 5 years and 11 years and 11 months, possess auditory thresholds within normal limits across all frequencies bilaterally, have no conductive impairment at the time of the research, and have no prior auditory training or musical education. Additionally, participants were required not to be bilingual, not have previously diagnosed neurological or neurodevelopmental disorders such as autism spectrum disorder or attention deficit disorder with or without hyperactivity, not have school difficulties as reported by guardians, not require educational support at school, or have a history of retention. Furthermore, participants should not have had inadequate responses on central auditory processing (CAP) behavioral tests or frequency following response (FFR) records under non-ideal conditions.

The sample selection procedures were as follows:

1. Conducting an initial interview with the guardians to identify any impairments that might prevent the execution of the subsequent research procedures.
2. Meatoscopy using an otoscope (Mikatos, Brazil) aimed at ruling out potential external ear impairments that could interfere with the audiological assessment.
3. Threshold tone audiometry, covering frequencies of 250 Hz to 8 kHz. Children up to seven years old should have auditory thresholds lower than 15 dBHL across all evaluated frequencies^(21)^, while for children older than seven, hearing thresholds should be lower than 20 dBHL^(22)^.
4. Tympanometry, where participants should exhibit a type A tympanometric curve^(23)^.

The audiological examinations, as well as the assessment of CAP skills, were performed in an acoustic booth. An audiometer (AD229e, Interacoustics, Denmark) with supra-aural headphones (TDH 39, Telephonics, USA) was used. Acoustic immittance measurements (tympanometry) were obtained using an immittance meter (AZ26, Interacoustics, Denmark), with a test tone of 226 Hz employed. Notably, the equipment and acoustic booth were calibrated as outlined by ANSI S3.6, IEC 60645-1, and ISO 8253-1 standards.

Regarding the specific procedures of the study, a behavioral screening of CAP was conducted, focusing on binaural integration and auditory skills, as well as temporal resolution. This involved the application of the Auditory Processing Domains Questionnaire (APDQ)^(24)^ and the recording and analysis of the FFR.

In relation to the CAP screening, two tests were conducted concerning the auditory skills of binaural integration and temporal resolution. This choice was based on national and international recommendations^(25,26)^, which also included non-verbal response tests, considering that the study population presented with SSD.

To evaluate binaural integration skills, the dichotic digit test was administered at an intensity of 50 dB SL. The normality criterion established for children aged 5-6 years was ≥81% accuracy in the right ear and ≥74% in the left ear. For children aged 7-8 years, the minimum percentage of correct responses should be ≥85% for the right ear and ≥82% for the left ear. For those aged 9-11 years or older, accuracy percentages of ≥95% for both ears were considered within the normal range^(27)^.

To assess temporal resolution auditory skills, the random gap detection test was utilized, also at an intensity of 50 dB SL. The normality criteria established require that, for children aged 5-6 years, the average across four frequencies should be ≤15 ms, while for children aged >7 years, the average should not exceed 10 ms^(28)^.

To meet all ethical considerations and ensure transparency for all participants, an assessment report containing the CAP test scores, according to the normality criteria for the specific age group of each participant, was sent, and adequate intervention was offered if any deviation was detected.

Regarding the analysis of auditory behavior, the APDQ was applied^(24)^, which consists of 52 questions distributed across three domains: auditory processing, attention, and language. Responses are scored as follows: four points (>75%) when the behavior occurs almost always; three points (>44%) if observed frequently; one point (<44%) for sometimes, and zero points (<25%) if the behavior was rarely observed. The scores in each domain were presented as percentages. The collected data were recorded in an Excel spreadsheet provided by the questionnaire authors, where domain scores were calculated. The higher the score, the better the auditory behavior is perceived by the parents and/or guardians.

In order to analyze the neural encoding of speech sounds, it was first necessary to analyze the FFR. The examination was conducted using the SmartEP module of Intelligent Hearing Systems, with the child seated comfortably with their eyes closed in a reclining chair in a quiet room. Before placing the surface electrodes, the skin was cleaned with abrasive gel (Nuprep^®^). Reference electrodes were fixed on the left mastoid (M1) and right mastoid (M2), and the active (Fpz) and ground electrodes (Fz) were positioned on the forehead, following the International Electrode System (IES 10-20) standard^(29)^ using electro-conductive paste and micropore adhesive tape.

The stimulus used was the synthetic syllable /da/ (40 ms), presented monaurally to the right ear using ER 3A insert earphones, at an intensity of 80 dB HL, with alternating polarity and a presentation rate of 10.9 ms. A 40 ms pre-stimulation was applied, with a 60 ms analysis window and a gain of 150 k. Low- and high-pass filters of 100 and 3000 Hz, respectively, were used. Impedance was maintained below 3 kΩ, ideally without differences between channels. Two stimulations of 3000 stimuli each were performed, combined post-collection to generate the resulting wave. Within this process, waves V, A, C, D, E, F, and O were marked^(30-32)^. Examinations exceeding a 10% artifact rate or ±35 μV were excluded.

Data analysis was conducted in the time domain, including absolute latency values (ms) (V, A, C, D, E, F, and O), slope measurement (ms/µV), component amplitude (µV), and displacements (ms) (V-D, C-D, D-O, A-D, and A-O).

The wave marking in the FFR time domain (ms) was done by two experienced speech therapists to minimize potential research biases, thus enabling more reliable further statistical testing. Inferential analysis of reliability (intraclass correlation coefficient [ICC]) and concordance employed the ICC functions from the IRR package in the R software. For this, reliability and concordance concerning latency variability were observed across all markings. For component V, reliability was excellent, with high concordance between evaluators (*p* < 0.05; ICC > 0.90). For component A, good reliability and high concordance were observed (*p* < 0.05; ICC = 0.75-0.90). For waves C and D, moderate reliability and good concordance were observed (*p* < 0.05; ICC = 0.50-0.75). Nevertheless, components E and F showed excellent reliability and high concordance between evaluators (*p* < 0.05; ICC > 0.90). For wave O, good reliability and high concordance were observed (*p* < 0.05; ICC = 0.75-0.90).

A basic frequency analysis of the FFR using the time-frequency distribution (TFD) of the spectrogram, available in the cABR software of the equipment, was also conducted. This approach enabled the illustration of neuronal activation in neurophysiological responses, as full frequency analysis was not available on the equipment used for this study.

All procedures were performed in the same sequence for both groups and on a single day.

Qualitative results of behavioral assessment and the APDQ were analyzed according to frequency and compared using Fisher’s exact test. After testing for normality assumptions (Shapiro-Wilk test), homogeneity of variances (Levene’s test), and independence of errors (residual analysis), because the variables latency, amplitude, displacement, and slope did not meet the assumptions, they were analyzed using non-parametric statistics, specifically the Mann-Whitney test. The correlation between behavioral tests and FFR latency results in the children of each group was performed using the Kendall method. All analyses were conducted using the R software. Significant differences were considered when *p* < 0.05.

## RESULTS

Regarding the sample composition, initially, 70 children were evaluated, of whom 42 were excluded because they did not meet the inclusion criteria. The groups consisted of 13 children in the EG diagnosed with PDs (aged 5-10 years, with a mean age of 7.0; four females and nine males) and 15 children in the CG with typical speech acquisition/development (nine females and six males), matched by age with the study group.

In the assessment of behavioral auditory skills of binaural integration and temporal resolution, as well as in the analysis of parent and/or guardian perception, qualitative data (normal or altered in the tests used) were analyzed in terms of frequency and compared between the groups studied using Fisher’s exact test.

Regarding the behavioral evaluation of auditory skills, a statistically significant difference was observed in the responses of the dichotic digit test (*p* < 0.01), indicating that children with PDs exhibit alterations in binaural integration auditory skills. In this test, the number of children with altered parameters was greater than expected (*p* < 0.01).

The random gap detection test and the APDQ results had no statistically significant differences (*p* > 0.05). In the descriptive analysis of the APDQ for the EG, among the six subjects with altered results, one was at risk for attention deficit hyperactivity disorder (ADHD), while three showed suspected CAP disorder, and another three showed suspected language impairment. In the CG, among the four participants with alterations, three were classified with a possible risk of having ADHD, and one showed a combination of risk for ADHD and CAP disorder.

For the analysis of neural encoding of speech sounds, the amplitude (µV), latency (ms), shift (ms), and slope (µV/ms) values measured in the FFR were compared between groups using the Mann-Whitney test.

For amplitude, there was no statistically significant difference between groups, albeit the EG demonstrated significantly higher latency values in the O component than the CG (*p* > 0.05). Other latency comparisons were not affected by the evaluated group.

One aspect requiring special attention is the result of the shift analysis, as the A-O shift was greater for the EG than the CG (*p* > 0.05). Other results related to shifts and slope showed no significant differences between the evaluated groups (*p* > 0.05).

To more thoroughly describe the relationship between changes in binaural integration and temporal resolution auditory skills in the EG, a correlation analysis was conducted between the behavioral tests and the latency (ms) results of the FFR using Kendall’s method. However, no significant correlation was observed between the behavioral tests and the latency results (*p* > 0.05).

The correlation between the severity of the PDs and the latencies of the different FFR components was also investigated using Kendall’s method. Only a significant positive correlation was identified between the severity of the PDs and the latency of the E component (*p* < 0.05; *r* = 0.56), suggesting that the more severe the disorder, the greater the E latency. Lastly, to qualitatively illustrate the neural encoding among the evaluated groups, the spectrogram (i.e., the TFD) obtained from the SmartEP software of one participant from each group is included.

**Figure 1:**
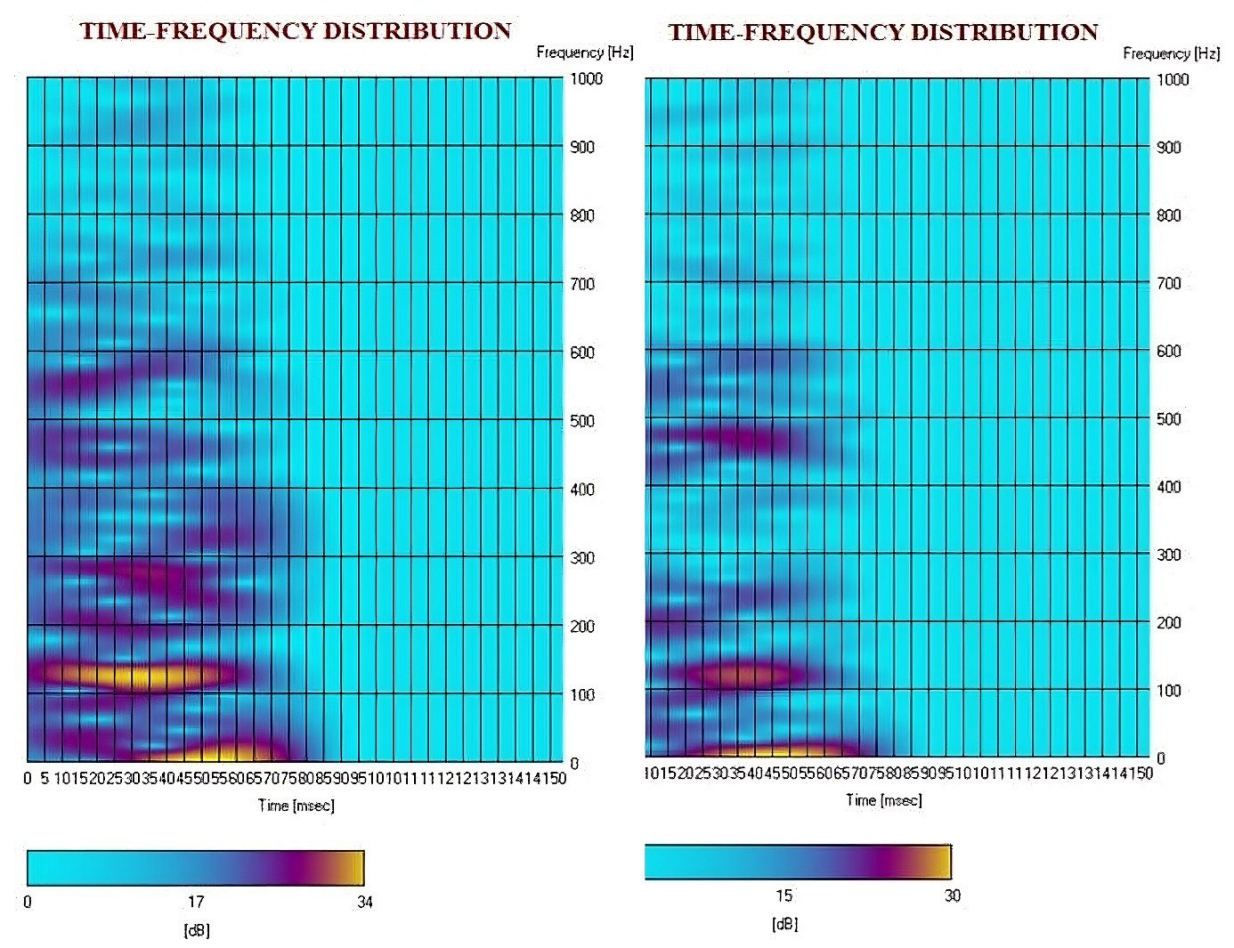
Illustration of the spectral graphical representation of the frequency following response from a participant in the control group (first plot) and a participant in the experimental group (second plot) using the SmartEP equipment

By analyzing the TFD in Figure 1, one can observe the differences when comparing the neurophysiological responses of the subject from the CG (in the first plot) with the subject from the EG (in the second plot). In the first plot, greater activation is evident in the 0-200 Hz range, as indicated by the expansion of the yellow color, as well as increased neuronal activation at higher frequencies. This phenomenon was not observed in the child from the EG, where there is practically no neurophysiological manifestation. It is worth noting that these children were randomly selected from both groups to illustrate the spectral differences in neural encoding.

## DISCUSSION

Dynamic models aiming to analyze the development of speech production emphasize the interdependence between auditory perception, production, and sound representation^(2-4)^. Thus, it is inferred that alterations in central auditory skills may hinder the formation of phonemic representation at the cortical level, thereby interfering with the learning of phonological, syntactic, and semantic rules^(33)^. Furthermore, children experiencing PDs and/or CAP disorders often exhibit a higher occurrence of various phonological processes, which frequently render speech unintelligible^(3-5,17)^.

This study provides further evidence of this interaction, particularly considering the occurrence of alterations in binaural integration auditory skills in children with SSD, as also reported elsewhere^(4,5,17)^. This auditory skill plays a critical role in speech perception and production. Therefore, any alteration in this auditory skill may have a negative impact, interfering with the perception of speech sounds^(6)^, as observed in our study.

Additionally, dichotic tests (i.e., the dichotic digits test) involve a key central nervous system structure—the corpus callosum. This structure plays an important role in the integration between the right and left hemispheres, as its fibers connect to central auditory pathways. Dichotic tests can assess the functioning of this structure by executing the task of binaural integration. An alteration in these tests may indicate difficulty in transferring information from the right hemisphere to the left^(25,34,35)^. In this regard, in binaural integration, the EG showed a higher likelihood of alteration (Table 1).

**Table 1:** Responses to behavioral tests and the Auditory Processing Domains Questionnaire as per the study groups (n = 28)

Central auditory processing test	n (%)		*p-*value
	Control (n = 15)	Experimental group (n = 13)	
Binaural integration dichotic digit test			
Normal	8 (28.57)	0 (0.0)	< 0.01*
Altered	7 (25.00)	13 (46.43)	
Random gap detection test			
Normal	12 (42.85)	7 (25.00)	0.23
Altered	3 (10.71)	6 (21.43)	
Auditory Processing Domains Questionnaire			
Normal	11 (39.28)	6 (21.42)	0.24
Altered	4 (14.28)	7 (25.00)	

* Probability by Fisher’s exact test at 5% significance (statistically significant value)

Therefore, one can infer that the dichotic listening process in children is still developing. Children experiencing SSD of idiopathic origin or associated with neurodevelopmental disorders may present dysfunctions, suggesting an influence on the maturation process of the CANS and, consequently, on the development of auditory skills.

Previous studies have reported that children diagnosed with SSD exhibit changes in temporal auditory processing^(36,37)^. It is known that children with SSD require a longer time interval to perceive differences between sounds^(38,39)^. However, in our study, it was not possible to confirm such a difference, possibly due to the difficulty in finding children without altered auditory skills in the CG. This observation is also corroborated by the APDQ results, wherein, regardless of the group, parents and/or guardians reported changes in their dependents’ auditory behavior.

In regard to the APDQ, alterations were distributed among risks of ADHD, suspected CAPD, and suspected language disorders in the EG. In the CG, most alterations were related to ADHD, with one case associated with a combination of ADHD and CAPD. Thus, it is plausible to suggest that the characteristics of the EG may be associated with a greater diversity of difficulties, whereas ADHD was more predominant in the CG.

These findings suggest that the relationship between ADHD, CAPD, and language difficulties may vary according to the characteristics of the EG, emphasizing the need for multidisciplinary approaches and detailed evaluations to differentiate between auditory and attentional difficulties in the identification and intervention of these disorders. Moreover, the suspected alterations in both groups highlight the importance of early screening and differential diagnosis, ensuring more effective follow-up strategies.

The importance of using self-perception questionnaires, applied to parents and/or guardians, regarding children’s auditory behavior, in matters concerning CAPD, is emphasized^(40)^. The APDQ^(24)^, the instrument used in this study, was recently validated for Brazilian Portuguese, highlighting the lack of research that employed its application in populations with suspected and/or altered CAPD. Indeed, the most commonly used questionnaire in research is the Scale of Auditory Behavior^(41)^, although it has only been validated for European Portuguese, which differs in sociocultural and economic aspects from its Brazilian variant. For this reason, we opted to apply the APDQ.

Furthermore, the APDQ consists of various questions and may prove complex for this specific population due to difficulties in fully understanding the questions, which may have interfered with the results. Nevertheless, no studies were found in the literature that enabled us to conduct a comparison with the data presented herein.

As for the neural encoding of speech, a statistically significant difference was only observed for component O of the FFR in the children with PD in the EG compared to the CG. However, for the most part, the latency values of the different components of the FFR in the PD group were higher than those of the CG. Regarding interpeak latencies, we observed that the A-O shift exhibited an increased value in the PD group (Table 2). This result partially corroborates a recent study involving 60 participants aged between 5 years and 8 years and 11 months and divided into two groups: 30 with typical speech development and 30 diagnosed with PDs. The authors concluded that children with PD exhibited altered neural encoding of speech sounds, as evidenced by increased latencies in all components of the FFR, with statistically significant differences in waves V, A, F, and O^(8)^.

**Table 2:** Comparison of amplitude (µV), latency (ms), shift (ms), and slope (µV/ms) of the frequency following response according to the study groups (n = 28)

Responses	Groups		1SEM	*p*-value
	Control (n = 15)	Experimental group (n = 13)		
Amplitude				
V	0.26	0.32	0.03	0.20
A	-0.16	-0.19	0.02	0.78
C	-0.16	-0.19	0.03	0.66
D	-0.23	-0.2	0.02	0.76
E	-0.23	-0.24	0.03	0.94
F	-0.2	-0.24	0.02	0.73
O	-0.23	-0.25	0.02	0.99
Latency				
V	6.78	6.87	0.06	0.56
A	7.86	8.06	0.07	0.27
C	17.19	17.19	0.2	0.99
D	23.32	23.08	0.3	0.20
E	31.71	32.39	0.45	0.94
F	40.48	40.82	0.25	0.63
O	48.33	49.22	0.27	0.04*
Shifts				
V-D	16.54	16.21	0.3	0.39
C-D	6.12	5.89	0.32	0.68
D-O	25.01	26.14	0.35	0.18
A-D	15.46	15.02	0.29	0.53
A-O	40.47	41.16	0.25	0.03*
Slope	0.4	0.49	0.04	0.61

* Probability by the Mann-Whitney test at 5% significance

Caption: SEM = Standard error of the mean

Wave O is characterized as the measure representing the end of the acoustic stimulus, reflecting notable and lasting changes throughout human brain development^(42)^. Thus, children with PD have compromised structures responsible for encoding at the end of a stimulus, specifically at the end of a syllable^(43)^. Hence, it is emphasized that this alteration directly impacts speech perception and consequently production, hindering articulatory precision and communication clarity. Additionally, it is plausible to suggest that the difficulty in processing syllable endings may influence the phonological construction of words, affecting the development of oral language and, later, the acquisition and development of written language.

Nevertheless, it is worth noting that latency measures may not be the most suitable for accurately representing the subcortical response, suggesting that frequency measures, rather than temporal measures, could be a more appropriate method for measuring neural encoding in children with PDs, as highlighted in another study^(44)^. Consequently, the differences in neural encoding between the evaluated groups were illustrated through the TFD analysis of a subject from the EG and a subject from the CG (Figure 1), as an example of this analytical possibility, which will be discussed further.

By analyzing the severity of PD and its relationship with latency values of the FFR components, we noted that the more severe the PD, the greater the latency, especially of component E. Together, waves D, E, and F are responsible for encoding the periodic and harmonic sound structure of the vowel /a/^(42,43)^. Thus, children with greater speech alterations may present an altered sustained portion, particularly in component E. This reinforces the hypothesis that deficits in neural encoding of speech sounds may be associated with difficulties in articulatory production. Since the sustained portion of the FFR is related to the stability of the neural representation of verbal sounds, alterations in this parameter may indicate compromised underlying auditory processing.

Moreover, deficits in the perception and maintenance of vowel sound structure can directly impact phonological acquisition and speech intelligibility, contributing to an atypical pattern of oral language development. These results highlight the importance of neurophysiological assessments (e.g., the FFR) in the early identification of auditory alterations underlying SSDs, as has already been demonstrated in the literature^(8,9)^. It is believed that understanding latency patterns and their implications in speech processing may guide more effective therapeutic approaches, favoring intervention strategies that optimize the perception and production of speech sounds in children with PDs. Nevertheless, the initial hypothesis was that the severity of PD would be directly proportional to the increase in latency of all the components measured in the FFR.

Regarding the amplitude of the FFR components and the slope value, which reflects the relationship between time (ms) and magnitude (µV) of the neural response of the VA complex^(43)^, we did not identify significant statistical differences between the groups, contradicting the initial hypothesis. Analyzing possible explanations for these results, especially when comparing them with those of a study^(8)^ that investigated children with PDs and observed differences in latencies of other FFR components (V, A, F, and O), as well as in the slope, it is plausible to suggest that the discrepancy may be attributed to the CG. During the data collection period of this study in 2023 (i.e., the post-COVID-19 period), significant obstacles were encountered in identifying children with altered auditory behavioral skills. Therefore, normal results in both CAP tests were not considered for inclusion in the criteria, given that the primary focus was to investigate binaural integration and temporal resolution auditory skills, along with neural encoding in children with PDs, regardless of the CAP status of children with typical speech.

In the simplified spectrogram analysis, visually examining each record of the different sample subjects revealed differences in neuronal excitation between the studied groups. The predominance of the yellow color, indicating greater activation at 0-200 Hz in the CG individual, suggests that these manifestations may be related to significant behavioral alterations detected through electrophysiology, indicating lower neuronal activation in the EG, which is composed of children with PDs. This frequency range is directly associated with the human vocal tone and phase-locking activity^(45)^. Furthermore, various researchers have highlighted the relevance of the FFR in understanding the encoding of sound information, both subcortically and cortically, pointing to differentiation in responses according to the neural centers involved in each pathology^(46)^.

Notably, the spectrogram analysis was conducted solely for didactic purposes and to enhance the visualization of neurophysiological activations, as only one randomly selected participant from each group was considered in the preparation of Figure 1. It is suggested that no generalizations and/or further inferences be made considering such a comparison. Nevertheless, this data indicates another potential research pathway with the FFR in children with SSDs, especially those with PDs.

This study reinforces, once again, the importance of hearing for proper speech production, justifying the inclusion of auditory skills stimulation activities within the therapeutic context for children with PDs, contributing to a more comprehensive, focused, and effective therapeutic approach. This clinical practice is promising, potentially even reducing the duration of speech-language intervention, which represents an advantage for both private and especially public services, burdened by the high demand for care for children with SSD.

The main limitation of this study may lie in the composition of the CG. Future investigations into other auditory skills would be relevant for a more comprehensive understanding of how the CANS of these children behaves. Lastly, the importance of a more detailed frequency analysis of speech neural encoding in children with SSD is highlighted.

## CONCLUSION

Children with PDs exhibited alterations in binaural integration auditory skills and in the neural encoding of speech sounds. Thus, both behavioral and electrophysiological results suggested a possible relationship between auditory perception and speech production in children with PDs.

## References

[1] Chang SE, Guenther FH (2020). Involvement of the cortico-basal ganglia-thalamocortical loop in developmental stuttering. Front Psychol.

[2] Guenther FH (2006). Cortical interactions underlying the production of speech sounds. J Commun Disord.

[3] Berti LC, Guilherme J, Esperandino C, Oliveira AM (2020). Relationship between speech production and perception in children with Speech Sound Disorders. J Port Linguist..

[4] Barrozo TF, Pagan-Neves LO, Vilela N, Carvallo RMM, Wertzner H (2016). The influence of (central) auditory processing disorder in speech sound disorders. Rev Bras Otorrinolaringol.

[5] Paz-Oliveira A, Momensohn-Santos TM, Branco-Barreiro FC (2015). Associação entre desvio fonológico e distúrbio do processamento auditivo central: revisão da literatura. Equilíbrio Corp Saúde..

[6] Westerhausen R, Hugdahl K (2008). The corpus callosum in dichotic listening studies of hemispheric asymmetry: A review of clinical and experimental evidence. Neurosci Biobehav Rev.

[7] Dias KZ, Jutras B, Acrani IO, Pereira LD (2012). Random Gap Detection Test (RGDT) performance of individuals with central auditory processing disorders from 5 to 25 years of age. Int J Pediatr Otorhinolaryngol.

[8] Ferreira L, Gubiani MB, Keske-Soares M, Skarzynski PH, Sanfins MD, Biaggio EPV (2019). Analysis of the components of Frequency-Following Response in phonological disorders. Int J Pediatr Otorhinolaryngol.

[9] Marques MCS, Griz S, de Andrade KCL, Menezes PL, Menezes DC (2021). Frequency Following Responses in childhood apraxia of speech. Int J Pediatr Otorhinolaryngol.

[10] Ceron MI, Gubiani MB, Oliveira CR, Gubiani MB, Keske-Soares M (2017). Ocorrência do desvio fonológico e de processos fonológicos em aquisição fonológica típica e atípica. CoDAS.

[11] ASHA: American Speech-Language-Hearing Association (2014). Speech sound disorders: articulation and phonological processes.

[12] Dodd B (2014). Differential diagnosis of pediatric speech sound disorder. Curr Dev Disord Rep.

[13] Bowen C. (2015). Children’s speech sound disorders..

[14] Shriberg LD, Campbell TF, Mabie HL, McGlothlin JH (2019). Initial studies of the phenotype and persistence of speech motor delay (SMD). Clin Linguist Phon.

[15] Dodd B (2011). Differentiating speech delay from disorder: does it matter?. Top Lang Disord.

[16] Rabelo ATV, Alves CRL, Goulart LMHF, Friche AAL, Lemos SMA, Campos FR (2011). Alterações de fala em escolares na cidade de Belo Horizonte. J Soc Bras Fonoaudiol.

[17] Brancalioni AR, Bertagnolli APC, Bonini JB, Gubiani MB, Keske-Soares M (2012). The relation between auditory discrimination and phonological disorder. J Soc Bras Fonoaudiol.

[18] Vilela N, Barrozo TF, Pagan-Neves LO, Sanches SGG, Wertzner HF, Carvallo RMM (2016). The influence of (central) auditory processing disorder on the severity of speech-sound disorders in children. Clinics.

[19] Shriberg LD, Kwiatkowski J (1982). Phonological disorders: I: A diagnostic classification system. J Speech Hear Disord.

[20] Shriberg LD, Austin D, Lewis BA, McSweeny JL, Wilson DL (1997). The percentage of consonants correct (PCC) metric: extensions and reliability data. J Speech Lang Hear Res.

[21] Northern JL, Downs MP (2002). Hearing in children..

[22] OMS: Organização Mundial da Saúde (2020). Prevention of blindness and deafness.

[23] Jerger J (1970). Clinical experience with impedance audiometry. Arch Otolaryngol.

[24] Dias KZ, Yokoyama CH, Pinheiro MMC, Braga J, Pereira LD, O’Hara B (2022). The Auditory Processing Domains Questionnaire (APDQ): Brazilian-Portuguese version. Rev Bras Otorrinolaringol.

[25] ABA: Academia Brasileira de Audiologia (2016). Recomendações e valores de referência para o protocolo de avaliação do PAC: comportamental e eletrofisiológica..

[26] ASHA: American Speech-Language-Hearing Association (2005). (Central) auditory processing disorders: the role of the audiologist. Position statement.

[27] Pereira L, Schochat E (2011). Testes auditivos comportamentais para avaliação do processamento auditivo central..

[28] Ziliotto K, Pereira LD (2005). Random Gap Detection Test in subjects with and without APD..

[29] Klem GH, Lüders HO, Jasper HH, Elger C (1999). The ten-twenty electrode system of the International Federation. Electroencephalogr Clin Neurophysiol Suppl.

[30] Gabriel LB, Vernier LS, Ferreira MIDC, Silveira AL, Machado MS (2018). Parameters for applying the brainstem auditory evoked potential with speech stimulus: a systematic review. Int Arch Otorhinolaryngol.

[31] Kraus N, Anderson A, White-Schwoch T, Fay RR, Popper AN (2017). The frequency-following response: a window into human communication..

[32] Sanfins MD, Garcia MV, Biaggio EPV, Skarzynski PH (2019). The frequency following response: evaluations in different age groups..

[33] Smith A, Hardcastle WJ, Laver J, Gibbon FE (2010). The handbook of phonetic sciences..

[34] McArthur GM, Bishop DV (2005). Speech and non-speech processing in people with specific language impairment: A behavioral and electrophysiological study. Brain Lang.

[35] AAA: American Academy of Audiology (2010). Clinical practice guidelines: diagnosis, treatment and management of children and adults with central auditory processing disorder.

[36] Stroiek S, Silva Quevedo L, Hernandez Kieling C, Lago Battezini AC (2015). Auditory training in auditory processing disorders: a case study. Rev CEFAC.

[37] Muniz LF, Roazzi A, Schochat E, Teixeira CF, Lucena JA (2007). Avaliação da habilidade de resolução temporal, com uso do tom puro, em crianças com e sem desvio fonológico. Rev CEFAC.

[38] Jain CH, Priya MB, Joshi K (2020). Relationship between temporal processing and phonological awareness in children with speech sound disorders. Clin Linguist Phon.

[39] Santos JLF, Parreira LMMV, Leite RCD (2010). Habilidades de ordenação e resolução temporal em crianças com desvio fonológico. Rev CEFAC.

[40] Souza IMP, Carvalho NG, Plotegher SDC, Colella-Santos MF, Ramos do Amaral MI (2018). Triagem do processamento auditivo central: contribuições do uso combinado de questionário e tarefas auditivas. Audiol Commun Res.

[41] Nunes CL, Pereira LD, Carvalho GS (2013). Scale of Auditory Behaviors e testes auditivos comportamentais para avaliação do processamento auditivo em crianças falantes do português europeu. CoDAS.

[42] Skoe E, Krizman J, Anderson S, Kraus N (2015). Stability and plasticity of auditory brainstem function across the lifespan. Cereb Cortex.

[43] Russo N, Nicol T, Musacchia G, Kraus N (2004). Brainstem responses to speech syllables. Clin Neurophysiol.

[44] Filippini R, Befi-Lopes DM, Schochat E (2012). Efficacy of auditory training using the auditory brainstem response to complex sounds: auditory processing disorder and specific language impairment. Folia Phoniatr Logop.

[45] Zhang X, Gong Q (2017). Correlation between the frequency difference limen and an index based on principal component analysis of the frequency-following response of normal hearing listeners. Hear Res.

[46] Coffey EBJ, Nicol T, White-Schwoch T, Chandrasekaran B, Krizman J, Skoe E (2019). Evolving perspectives on the sources of the frequency-following response. Nat Commun.

