# Supplementary material for: Auditory skills and neural encoding in children with speech sound disorder
Source: Codas. 2025 Nov 17;37(6):e20240173. doi: 10.1590/2317-1782/e20240173en (PMC12642776; doi:10.1590/2317-1782/e20240173en)
Supplement: Translation

# Translation: Habilidades auditivas e codificação neural em crianças com transtorno dos sons da fala

**DOI:** 10.1590/2317-1782/e20240173pt

**Authors:** Dara Eliza Rohers, Márcia Keske-Soares, Eliara Pinto Vieira Biaggio

**Affiliations:** 1 Departamento de Fonoaudiologia, Universidade Federal de Santa Maria – UFSM - Santa Maria (RS), Brasil.

**Keywords:** Distúrbios da Fala, Eletrofisiologia, Processamento Auditivo, Criança, Percepção da Fala, Transtornos da Articulação

## Abstract

**Objetivo:**

Investigar as habilidades auditivas de integração binaural e resolução temporal, mensurar a percepção de pais e/ou responsáveis quanto ao comportamento auditivo de seus dependentes, além de analisar a codificação neural em crianças com Transtorno Fonológico (TF).

**Método:**

Participaram do estudo 28 crianças, divididas em dois grupos: 13 com TF (média de idade de 7:0 anos) e 15 crianças com desenvolvimento típico da fala, emparelhados por idade com o grupo estudo. Avaliou-se as habilidades auditivas de integração binaural e resolução temporal. Pais e/ou responsáveis preencheram o questionário Auditory Processing Domains Questionnaire (APDQ). A codificação neural foi analisada utilizando-se o Frequency Following Response (FFR) com estímulo /da/, avaliando-se as amplitudes, latências absolutas, deslocamentos, e a medida do slope. Para a análise frequencial básica do FFR, empregou-se o Time-Frequency Distribution (TFD) do espectrograma. A análise inferencial dos dados foi conduzida.

**Resultados:**

Foram observadas diferenças estatisticamente significativas na habilidade auditiva de integração binaural. Porém, o mesmo não foi visualizado na resolução temporal. Os pais e/ou responsáveis reportaram alterações no comportamento auditivo de seus dependentes em ambos os grupos. Já, na análise da codificação neural, as crianças com TF apresentaram latência mais elevada no componente O, com maior deslocamento A-O. Houve correlação positiva entre a gravidade do TF e a latência do componente E. A análise do espectrograma revelou maior excitação neuronal no grupo com desenvolvimento típico.

**Conclusão:**

Crianças diagnosticadas com TF apresentam alterações na habilidade auditiva de integração binaural e na codificação neural dos sons da fala.

## INTRODUÇÃO

A produção de fala envolve redes corticais e subcorticais sensoriais e motoras no cérebro, facilitando a integração de informações auditivas, a representação dos sons e, consequentemente, o planejamento e execução de atos motores para a emissão dos sons de fala^(1,2)^. Portanto, apenas a sensibilidade auditiva não é suficiente para o desenvolvimento da percepção e da produção da fala, pois é crucial que as informações auditivas recebidas sobre sons complexos sejam interpretadas de maneira eficaz^(3)^.

Entender como o Sistema Nervoso Auditivo Central (SNAC) processa e utiliza a informação recebida é fundamental para compreender como as alterações de fala se manifestam em crianças com Transtorno dos Sons da Fala (TSF), especialmente no Transtorno Fonológico (TF)^(4,5)^.

Destacam-se, as habilidades auditivas de integração binaural e resolução temporal, as quais desempenham um papel importante no Processamento Auditivo Central (PAC) e, por extensão, na fala. Na integração binaural, as informações auditivas recebidas são enviadas para os hemisférios cerebrais por meio do corpo caloso, permitindo a interpretação desses sinais acústicos. Esse processo facilita a identificação de diferenças sutis nos sons, além de permitir a distinção de sons sobrepostos em ambientes ruidosos, como o som da fala em meio ao barulho. Ressalta-se ainda que essa estrutura altamente mielinizada possui fibras de todas as modalidades sensoriais, além de seu envolvimento na modulação da atenção^(6)^.

Por outro lado, a resolução temporal diz respeito à capacidade de perceber mudanças rápidas na duração e/ou interrupções em estímulos auditivos. Essa habilidade engloba também a percepção de diferenças entre sons produzidos no mesmo ponto articulatório, distinguíveis apenas pela sonorização. É fundamental destacar que a resolução temporal é essencial no reconhecimento dos sons da fala, na percepção de variações na duração, das pausas e na velocidade das sílabas^(7)^.

No que tange à avaliação neurofisiológica da via auditiva, os Potenciais Evocados Auditivos com estímulo de fala proporcionam uma análise mais robusta do SNAC, especialmente na avaliação da codificação neural dos sons verbais associados às habilidades auditivas e, consequentemente, à comunicação. Nesse contexto, o *Frequency Following Response* (FFR) reflete o funcionamento das regiões corticais e subcorticais relacionadas à produção de fala^(8,9)^.

Analisando aspectos específicos da produção de fala, observa-se que a aquisição fonológica no processo de desenvolvimento linguístico é gradual e ocorre conforme a criança cresce, culminando na produção adequada de todos os sons da fala. No Português Brasileiro, o inventário fonológico completa-se por volta dos cinco anos de idade^(10)^.

Contudo, algumas crianças podem ter dificuldades de serem compreendidas verbalmente quando o desenvolvimento da fala não ocorre como esperado ou segue um padrão atípico, o que pode levar a diferentes TSF. Esses transtornos podem incluir uma variedade ou combinação de dificuldades de percepção, produção motora e/ou representação fonológica dos segmentos da fala e seus aspectos prosódicos^(11-15).^

O TF, o mais prevalente dos TSF na prática fonoaudiológica, caracteriza-se pela dificuldade da criança em pronunciar corretamente as palavras, apresentando principalmente omissões e/ou substituições de consoantes e em encontros consonantais^(16,17)^ o que resulta em fala ininteligível. Portanto, entender a relação das habilidades auditivas e linguísticas tem sido um desafio para muitos pesquisadores, sendo que existem evidências da necessidade de avaliação do processamento da informação nas vias auditivas centrais, para além das questões puramente de fala nas crianças com TF^(2,4,17,18)^.

Assim, investigar as habilidades auditivas relacionadas à produção da fala e sua relação com a codificação neural torna-se interessante, a fim de contribuir para o delineamento do processo terapêutico com ênfase não apenas nas questões de fala, mas abrangendo as questões auditivas das crianças com TF. Uma vez que, quanto maior o esclarecimento do papel das informações auditivas na produção de sons da fala e a compreensão de como a percepção auditiva interage com os sistemas motores e sensoriais^(2)^ pode-se aperfeiçoar o planejamento das estratégias de terapia fonoaudiológica nas crianças com TF.

A partir do exposto, este estudo teve como objetivo analisar as habilidades auditivas de integração binaural e resolução temporal, mensurar a percepção dos pais e/ou responsáveis quanto ao comportamento auditivo de seus dependentes, além de investigar a codificação neural em crianças com TF.

## MÉTODO

Esta pesquisa, de delineamento transversal, analítico e quantitativo, foi aprovada pelo Comitê de Ética em Pesquisa de uma instituição pública de ensino superior, sob o número 68074623.0.0000.5346. O estudo respeitou todas as normas e diretrizes regulamentadoras para pesquisa do Conselho Nacional de Saúde do Brasil. A amostra foi dividida em dois grupos: o grupo experimental (GE), composto por crianças diagnosticadas com TF, e o grupo controle (GC), composto por crianças com aquisição/desenvolvimento de fala típico.

O diagnóstico de fala para as crianças do GE foi realizado antecipadamente por uma equipe qualificada, fundamentando-se em avaliações de habilidades léxico-semânticas e memória de longo prazo na linguagem expressiva, vocabulário receptivo, avaliação fonológica por meio do INFONO, habilidades motoras de fala e Avaliação Miofuncional Orofacial com Escores. Baseando-se nos dados obtidos na avaliação fonológica, particularmente na etapa de nomeação espontânea, a gravidade do TF foi determinada pelo Percentual de Consoantes Corretas-Revisado (PCC-R), que se classifica em quatro níveis: leve (PCC > 85%), leve-moderado (PCC = 65-85%), moderado-grave (PCC = 50-65%), e grave (PCC < 50%)^(19,20)^. O GE consistiu em crianças em lista de espera para iniciar a reabilitação em um serviço de atendimento fonoaudiológico.

Para formar o GC, recrutaram-se crianças de forma conveniente, utilizando a técnica de amostragem “bola de neve”. As crianças do GC não foram submetidas a todas as avaliações mencionadas anteriormente, mas durante entrevistas com os pais, apurou-se que elas nunca haviam realizado tratamento fonoaudiológico para a fala, a família não tinha queixas nem reconhecia atraso no desenvolvimento da fala do(a) filho(a) em comparação com seus pares. Além disso, embora em uma observação não instrumental, a pesquisadora responsável não detectou nenhuma atipia na produção oral da criança avaliada.

Como critérios de elegibilidade para ambos os grupos, estipulou-se que as crianças deveriam ter entre cinco anos e 11 anos e 11 meses; apresentar limiares auditivos dentro da normalidade em todas as frequências e de forma bilateral; não ter comprometimento condutivo no momento da pesquisa; não ter passado por treinamento auditivo prévio, não ter recebido educação musical, não ser bilíngue, não apresentar transtornos neurológicos ou do neurodesenvolvimento diagnosticados previamente, como Transtorno do Espectro Autista e Transtorno do Déficit de Atenção com ou sem Hiperatividade, não ter dificuldades escolares auto relatadas pelos responsáveis, não necessitar de apoio educacional na escola e/ou ter historial de reprovação e não ter apresentado respostas inadequadas nos testes comportamentais de PAC ou registros do FFR sem condições ideais.

Os procedimentos de seleção da amostra foram os seguintes:

1. Realização de uma entrevista inicial com os responsáveis para identificar a presença de algum comprometimento que pudesse impedir a execução dos demais procedimentos da pesquisa.
2. Meatoscopia utilizando um otoscópio (Mikatos, Brasil)*,* com o objetivo de descartar eventuais comprometimentos da orelha externa que pudessem interferir na avaliação audiológica.
3. Audiometria tonal liminar, que abrangeu frequências de 250 Hz a 8 kHz. As crianças de até sete anos deveriam apresentar limiares auditivos inferiores a 15 dBNA em todas as frequências avaliadas^(21)^, enquanto para as crianças maiores de sete anos, os limiares auditivos deveriam ser inferiores a 20 dBNA^(22)^.
4. Timpanometria, na qual os participantes deveriam apresentar uma curva timpanométrica do tipo A^(23)^.

Os exames audiológicos, bem como a avaliação das habilidades do PAC, foram executados em uma cabine acústica. Utilizou-se um audiômetro (AD229e, *Interacoustics*, Dinamarca) com fones supra-aurais (TDH 39, *Telephonics*, EUA). As medidas de imitância acústica (timpanometria) foram obtidas com o uso de um imitanciômetro (AZ26, Interacoustics, Dinamarca), empregando um tom de teste de 226 Hz. Importante destacar que a calibração dos equipamentos e da cabine acústica estava em conformidade com as normas ANSI S3.6, IEC 60645-1 e ISO 8253-1.

Quanto aos procedimentos específicos da pesquisa, realizou-se o rastreio comportamental do Processamento Auditivo Central, focando na habilidade auditiva de integração binaural e na resolução temporal, aplicação do questionário *Auditory Processing Domains Questionnaire* (APDQ)^(24)^ além do registro e análise do FFR*.*

Em relação ao rastreio comportamental do PAC, foram realizados dois testes relacionados às habilidades auditivas de integração binaural e à resolução temporal. Esta escolha baseou-se em recomendações nacionais e internacionais^(25,26)^ incluiu também testes com respostas não verbais, considerando que a população do estudo apresentava TSF.

Para avaliar a habilidade integração binaural, foi aplicado o Dicótico de Dígitos (DD) na intensidade de 50dB NS. O critério de normalidade estabelecido para crianças de cinco a seis anos foi de ≥81% de acertos na orelha direita (OD) e de ≥74% na orelha esquerda (OE). Para as crianças de sete a oito anos, a porcentagem mínima de acertos deveria ser de ≥85% para a OD e de ≥82% para a OE. Já para aquelas de 9 a 11 anos ou mais, considerou-se dentro do padrão de normalidade uma porcentagem de acerto de ≥95% para ambas as orelhas^(27).^

Para avaliar a habilidade auditiva de resolução temporal, optou-se pelo teste *Random Gap Detection Test* (RGDT) que foi aplicado na intensidade de 50dB NS. Os critérios de normalidade estabelecem que, para crianças de cinco a seis anos, a média das quatro frequências deve ser menor ou igual a 15ms, enquanto para crianças de sete anos ou mais, a média deve ser menor ou igual a 10ms^(28).^

Como forma de atender a todas as questões éticas e garantir a transparência para todos os participantes do estudo, enviou-se um relatório de avaliação contendo a pontuação obtida nos testes do PAC, de acordo com o critério de normalidade para a respectiva faixa etária de cada participante, e ofereceu-se uma intervenção adequada em caso de qualquer alteração detectada.

Quanto à análise do comportamento auditivo, aplicou-se o questionário APDQ^(24)^ que é composto por 52 perguntas distribuídas em três domínios: processamento auditivo, atenção e linguagem. As respostas são pontuadas da seguinte maneira: quatro pontos (>75%) quando o comportamento ocorre quase sempre; três pontos (>44%) se observado frequentemente; um ponto (<44%) se ocorre às vezes e zero pontos (<25%) se o comportamento foi observado raramente. As pontuações em cada domínio foram apresentadas em percentual. Os dados coletados foram registrados em uma planilha Excel fornecida pelos autores do questionário, na qual se calcularam os escores de cada domínio. Quanto maior a pontuação, melhor é considerado o comportamento auditivo, segundo a percepção dos pais e/ou responsáveis.

Para a análise da codificação neural dos sons da fala, procedeu-se ao estudo e análise do FFR. O exame foi realizado utilizando o módulo *SmartEP* da *Intelligent Hearing Systems*, com a criança acomodada confortavelmente, sentada de olhos fechados em uma poltrona reclinável, em uma sala silenciosa. Antes da colocação dos eletrodos de superfície, realizou-se a higienização da pele com gel abrasivo (Nuprep^®^). Os eletrodos de referência foram fixados na mastóide esquerda (M1) e na mastoide direita (M2), e os eletrodos ativo (Fpz) e terra (Fz) foram posicionados na testa, conforme o padrão *International Electrode System* (IES 10-20)^(29)^ utilizando pasta eletrolítica e fita adesiva microporosa.

O estímulo utilizado foi a sílaba sintética /da/ (40ms), apresentada de forma monoaural na orelha direita utilizando fones de inserção ER 3A, na intensidade de 80dB NA, com polaridade alternada e uma taxa de apresentação do estímulo de 10,9ms. Uma pré-estimulação de 40ms foi aplicada, janela de análise foi de 60ms e ganho de 150k. Foram utilizados filtros passa-baixa e passa-alta de 100Hz e 3000Hz, respectivamente. A impedância foi mantida abaixo de 3kΩ, idealmente sem diferenças entre os canais. Realizaram-se duas estimulações de 3.000 estímulos cada, que, após a coleta, foram somadas para gerar a onda resultante. Neste processo, marcaram-se as ondas: V, A, C, D, E, F e O^(30-32)^. Exames que ultrapassaram a taxa de 10% de artefatos foram excluídos e/ou ±35μV.

A análise dos dados foi realizada no domínio do tempo, incluindo valores das latências absolutas (ms) (V, A, C, D, E, F e O), medida do slope (ms/µV), amplitude dos componentes (µV) e deslocamentos (ms) (V-D, C-D, D-O, A-D, A-O).

É importante ressaltar que a marcação das ondas no domínio do tempo (ms) do FFR foi realizada por dois juízes, Fonoaudiólogos, com experiência na realização do potencial. Esta etapa teve como objetivo minimizar possíveis vieses de pesquisa, permitindo, a partir dessa análise, avançar para os demais testes estatísticos com maior confiabilidade.

Na análise inferencial da confiabilidade (coeficiente de correlação intraclasse [CCI]) e da concordância, empregou-se as funções CCI do pacote IRR no software estatístico R. Neste caso, observou-se em relação à variável latência, confiabilidade e concordância entre todas as marcações. Para o componente V, a confiabilidade foi excelente, com alta concordância entre os avaliadores (*p* < 0,05; CCI > 0,90). Já para o componente A, foi observada boa confiabilidade e alta concordância entre os avaliadores (*p <* 0,05; CCI = 0,75-0,90). Para as ondas C e D, foi observada moderada confiabilidade e boa concordância (*p <* 0,05; CCI = 0,50-0,75). Já os componentes E e F apresentaram excelente confiabilidade e alta concordância entre os avaliadores (*p <* 0,05; CCI > 0,90). Para a onda O, foi observada boa confiabilidade e alta concordância entre os avaliadores (*p <* 0,05; CCI = 0,75-0,90).

Realizou-se, também, uma análise frequencial básica do FFR utilizando o *Time-Frequency Distribution* (TFD) do espectrograma, disponível no software cABR do equipamento. Esta abordagem permitiu uma ilustração da ativação neuronal nas respostas neurofisiológicas, visto que a análise frequencial completa não estava disponível no equipamento usado neste estudo.

Todos os procedimentos foram realizados na mesma sequência para ambos os grupos e em um único dia.

Os resultados qualitativos da avaliação comportamental e do APDQ foram analisados segundo a frequência e comparados pelo teste exato de *Fisher*. Após testadas as pressuposições de normalidade (teste de *Shapiro-Wilk*), homogeneidade das variâncias (teste de *Levene*) e independência dos erros (análise de resíduos), as variáveis latência, amplitude, deslocamento e inclinação (*slope*), por não atenderem às pressuposições, foram analisadas por estatística não paramétrica, utilizando-se o teste de *Mann-Whitney.* A correlação entre os testes comportamentais e os resultados da latência do FFR nas crianças de cada grupo foi realizada pelo método de *Kendall.* Todas as análises foram realizadas com o auxílio do programa estatístico R*.* Diferenças significativas foram consideradas quando *p* < 0,05.

## RESULTADOS

Quanto à composição amostral, inicialmente foram avaliadas 70 crianças, das quais 42 foram excluídas por não atenderem aos critérios de elegibilidade deste estudo. Assim, os grupos apresentaram uma composição final da seguinte maneira: o GE, composto por 13 crianças diagnosticadas com TF (idades entre 5 a 10 anos, com a média de idade de 7:0 anos, sendo quatro do sexo feminino e nove do sexo masculino) e GC, composto por 15 crianças com aquisição/desenvolvimento típico da fala (sendo nove do sexo feminino e seis do sexo masculino), emparelhados por idade com o grupo estudo.

Na avaliação das habilidades auditivas comportamentais de integração binaural e de resolução temporal, bem como na análise da percepção dos pais e/ou responsáveis, os dados qualitativos (normal ou alterado nos testes utilizados) foram analisados em termos de frequência e comparados entre os grupos estudados por meio do teste exato de *Fisher*.

Quanto à avaliação comportamental das habilidades auditivas, observou-se uma diferença estatisticamente significativa nas respostas do teste comportamental DD (*p <* 0,01), indicando que crianças com TF apresentam alterações na habilidade auditiva de integração binaural. Nesse teste, o número de crianças com parâmetros alterados foi maior que o esperado (*p <* 0,01).

O teste comportamental RGDT e a resposta ao questionário APDQ não apresentaram diferenças estatisticamente significativas (*p* > 0,05). Na análise descritiva do APDQ, observou-se que, no grupo estudo, dentre os seis sujeitos com resultados alterados, um participante apresentou risco para Transtorno do Déficit de Atenção e Hiperatividade (TDAH), enquanto três apresentaram suspeita de alteração no PAC e outros três, suspeita de alteração de linguagem. Já, no grupo controle, dentre os quatro participantes com alterações, três foram classificados com possível risco para TDAH, e um apresentou uma combinação de risco para TDAH e alteração no PAC.

Para análise da codificação neural dos sons da fala, compararam-se os valores de amplitude (µV), latência (ms), deslocamentos (ms) e inclinação (*slope*; µV/ms) mensurados no FFR entre os grupos avaliados, utilizando-se o teste de *Mann-Whitney.*

Em relação à amplitude, não se observou uma diferença estatisticamente significativa entre os grupos avaliados. No entanto, as crianças do GE demonstraram valores de latência significativamente mais altos no componente O em relação àqueles do GC (*p* > 0,05). As demais comparações de latência não foram afetadas pelo grupo avaliado.

Um aspecto que requer atenção especial é o resultado da análise dos deslocamentos, onde se observou que o deslocamento A-O foi maior para o GE em comparação ao GC (*p* > 0,05). Os demais resultados relacionados a deslocamentos e inclinação (*slope)* não apresentaram diferenças significativas em função do grupo avaliado (*p* > 0,05).

Para descrever de maneira mais aprofundada a relação entre mudanças nas habilidades auditivas de integração binaural e resolução temporal no GE, realizou-se a análise de correlação entre os testes comportamentais e os resultados de latência (ms) do FFR utilizando o método de *Kendall*. No entanto, não se observou nenhuma correlação significativa entre os testes comportamentais e os resultados de latência (*p* > 0,05).

A correlação entre a gravidade do TF e as latências dos diferentes componentes do FFR também foi investigada por meio do método de *Kendall.* Identificou-se apenas uma correlação positiva significativa entre a gravidade do TF e a latência do componente E (*p <* 0,05; r = 0,56), sugerindo que, quanto mais grave o TF, maior a latência de E. Por fim, para melhor ilustrar a codificação neural entre os grupos avaliados de forma qualitativa, optou-se por incluir a ilustração do espectrograma (ou seja, o TFD) disponível no *software Smart EP* de um sujeito de cada grupo, apenas para visualização didática.

Por meio da análise do TFD, apresentada na Figura 1 do espectrograma, foi possível observar diferenças ao comparar as respostas neurofisiológicas de um sujeito do grupo controle (representado na primeira figura) com um sujeito do grupo experimental (representado na segunda figura). Na primeira figura, nota-se uma maior ativação na faixa de 0-200 Hz, evidenciada pela expansão da cor amarela, além de uma maior ativação neuronal até mesmo em regiões de frequências mais elevadas. Tal fenômeno não é observado na criança do GE, na qual praticamente não há manifestação neurofisiológica. Cabe destacar que essas crianças foram selecionadas de maneira aleatória dentro dos dois grupos, com o objetivo de apresentar de forma didática as diferenças espectrais na codificação neural.

**Figura 1:** Ilustração da representação gráfica espectral do *Frequency Following Response* (FFR) de um indivíduo do grupo controle (primeiro traçado) e de um indivíduo do grupo experimental (segundo traçado) utilizando o equipamento SmartEP

## DISCUSSÃO

Os modelos dinâmicos, que buscam analisar o desenvolvimento da produção da fala, enfatizam a interdependência entre a percepção auditiva, a produção e a representação sonora^(2-4)^. Dessa forma, infere-se que alterações nas habilidades auditivas centrais podem dificultar a formação da representação fonêmica a nível cortical, interferindo, assim, na aprendizagem das regras fonológicas, da sintaxe e da semântica^(33)^. Ademais, destaca-se que crianças que vivenciam o TF e o Transtorno do Processamento Auditivo Central (TPAC) e/ou atraso das habilidades auditivas correlatas, frequentemente exibem uma maior ocorrência de diversos processos fonológicos, os quais muitas vezes tornam a fala ininteligível^(3-5,17).^

Este estudo acrescenta mais uma evidência dessa interação, especialmente considerando a ocorrência da alteração da habilidade auditiva de integração binaural nas crianças com TSF, resultado este também discutido por diversos pesquisadores^(4,5,17)^. Essa habilidade auditiva desempenha um papel fundamental na percepção e produção da fala. Portanto, qualquer alteração nessa habilidade auditiva pode ter um impacto negativo, interferindo na percepção apropriada e, consequentemente, na produção de sons da fala^(6)^ conforme observado nos dados do presente estudo.

Além disso, testes dicóticos, como o DD, envolvem a participação de uma estrutura crucial do sistema nervoso central — o corpo caloso. Tal estrutura tem um papel importante na integração entre os hemisférios direito e esquerdo, uma vez que suas fibras se conectam às vias auditivas centrais. Os testes dicóticos têm capacidade para avaliar o funcionamento dessa estrutura, por meio da execução da tarefa de integração binaural. Uma alteração nestes testes pode indicar uma dificuldade na transferência de informações do hemisfério direito para o esquerdo^(25,34,35)^. Nesse sentido, na integração binaural o GE apresentou maior probabilidade de alteração (conforme demonstrado na Tabela 1).

**Tabela 1:** Resposta aos testes comportamentais e ao questionário APDQ de acordo com os grupos estudados (n = 28 crianças)

Teste de PAC	n (%)		*p*
	Grupo controle (n = 15)	Grupo experimental (n = 13)	
DD IB			
Normal	8 (28,57)	0 (0,0)	<0,01^*^
Alterado	7 (25,00)	13 (46,43)	
RGDT			
Normal	12 (42,85)	7 (25,00)	0,23
Alterado	3 (10,71)	6 (21,43)	
APDQ			
Normal	11 (39,28)	6 (21,42)	0,24
Alterado	4 (14,28)	7 (25,00)	

* Probabilidade pelo teste exato de Fisher a 5% de significância (valor estatisticamente significante)

Legenda: PAC: Processamento Auditivo Central; DD: Dicótico de Dígitos; RGDT: *Random Gap Detection Test* (Teste de Detecção de Intervalo Aleatório); IB: Integração Binaural; APDQ: *Auditory Processing Domains Questionnaire*

Portanto, infere-se que o processo de escuta dicótica das crianças está em desenvolvimento e, possivelmente, crianças que vivenciam os TSF de origem idiopática ou associados a transtornos neurodesenvolvimentais podem apresentar uma disfunção, o que sugere uma influência no processo de maturação do SNAC e, consequentemente, no desenvolvimento das habilidades auditivas.

Em estudos anteriores observou-se que crianças diagnosticadas com TSF apresentavam alterações no processamento auditivo temporal^(36,37)^. É sabido que crianças com TSF necessitam de um intervalo de tempo maior para perceber diferenças entre sons^(38,39)^.No entanto, no presente estudo não foi possível confirmar tal diferença, provavelmente devido à dificuldade de se encontrar crianças sem alterações nas habilidades auditivas no GC. Esta observação também é reforçada pelas respostas ao questionário APDQ, no qual, independente do grupo, os pais e/ou responsáveis relataram alterações no comportamento auditivo de seus dependentes.

Entretanto, quanto ao APDQ, ressalta-se que no grupo estudo, as alterações foram distribuídas entre risco de TDAH, suspeita de alteração no PAC e suspeita de alteração de linguagem. Já no grupo controle, a maioria das alterações esteve relacionada ao TDAH, com um caso associado a uma combinação de TDAH e PAC. Dessa forma, sugere-se que as características do grupo estudo podem estar associadas a uma diversidade maior de dificuldades, enquanto no grupo controle o TDAH foi mais predominante.

Esses resultados sugerem que a relação entre TDAH, PAC e dificuldades de linguagem pode variar conforme as características do grupo avaliado, reforçando a necessidade de abordagens multidisciplinares e avaliações detalhadas para diferenciar dificuldades auditivas de dificuldades atencionais na identificação e intervenção desses transtornos. Além disso, a presença de suspeitas de alterações em ambos os grupos aponta para a relevância da triagem precoce e do diagnóstico diferencial, garantindo estratégias de acompanhamento mais eficazes.

Destaca-se a importância do uso de questionários de autopercepção, aplicados a pais e/ou responsáveis, em relação ao comportamento auditivo das crianças, nas questões referentes ao PAC^(40)^. O APDQ^(24)^ instrumento utilizado neste estudo, teve sua validação para o Português Brasileiro realizada recentemente, evidenciando a escassez de pesquisas que empregaram sua aplicação em populações com suspeita e/ou alteração de PAC. Ressalta-se que o questionário mais empregado em pesquisas é a *Scale of Auditory Behavior* ^(41)^ contudo, este é validado apenas para o Português Europeu, o qual difere em aspectos socioculturais e econômicos do Brasil. Por essa razão, optou-se, neste estudo, pela aplicação do APDQ.

Ademais, esse questionário é composto por diversas questões e pode se revelar complexo para essa população específica, devido à dificuldade de compreensão integral das questões, fato que pode ter interferido nas respostas. Não foram encontrados estudos na literatura que permitissem comparação com os dados aqui apresentados.

Sobre a codificação neural da fala, observou-se diferença estatisticamente significante apenas para o componente O do FFR nas crianças com TF no GE em comparação às crianças do GC. Entretanto, na maior parte, os valores de latência dos diferentes componentes do FFR no TF foram superiores aos do GC. Quanto às latências interpicos, notou-se que o deslocamento A-O apresentou valor aumentado no TF (conforme observado na Tabela 2). Esse resultado concorda, em parte, com um estudo recente que envolveu 60 participantes, com idades entre 5 anos e 8 anos e 11 meses, divididos em dois grupos: 30 com desenvolvimento típico de fala e 30 com diagnóstico de TF. As autoras concluíram que as crianças com TF apresentam alteração na codificação neural dos sons da fala, já que este grupo exibiu latências aumentadas em todos os componentes do FFR, com diferença estatisticamente significante nas ondas V, A, F e O^(8)^.

**Tabela 2:** Comparação da amplitude (µV), latência (ms), deslocamentos (ms) e *slope* (µV/ms) do *Frequency Following Response* de acordo com os grupos avaliados (n = 28 crianças)

Respostas	Grupos		1EPM	*p*
	Controle (n = 15)	Experimental (n = 13)		
Amplitude				
V	0,26	0,32	0,03	0,20
A	-0,16	-0,19	0,02	0,78
C	-0,16	-0,19	0,03	0,66
D	-0,23	-0,2	0,02	0,76
E	-0,23	-0,24	0,03	0,94
F	-0,2	-0,24	0,02	0,73
O	-0,23	-0,25	0,02	0,99
Latência				
V	6,78	6,87	0,06	0,56
A	7,86	8,06	0,07	0,27
C	17,19	17,19	0,2	0,99
D	23,32	23,08	0,3	0,20
E	31,71	32,39	0,45	0,94
F	40,48	40,82	0,25	0,63
O	48,33	49,22	0,27	0,04*
Deslocamentos				
V-D	16,54	16,21	0,3	0,39
C-D	6,12	5,89	0,32	0,68
D-O	25,01	26,14	0,35	0,18
A-D	15,46	15,02	0,29	0,53
A-O	40,47	41,16	0,25	0,03^*^
*Slope*	0,4	0,49	0,04	0,61

1 EPM = Erro padrão da média;

* Probabilidade pelo teste de Mann-Whitney a 5% de significância

A onda O é caracterizada como a medida que representa o término do estímulo acústico, refletindo alterações notáveis e duradouras ao longo do desenvolvimento do cérebro humano^(42)^. Assim, conclui-se que as crianças com TF apresentam comprometimento nas estruturas responsáveis pela codificação no final de um estímulo, ou seja, no final de uma sílaba^(43)^. Dessa forma, ressalta-se que essa alteração impacta diretamente na percepção e consequentemente na produção da fala, dificultando a precisão articulatória e a clareza da comunicação. Além disso, hipotetiza-se que a dificuldade em processar o final das sílabas possa influenciar na construção fonológica das palavras, afetando o desenvolvimento da linguagem oral e, posteriormente, a aquisição e desenvolvimento da linguagem escrita.

No entanto, é relevante salientar que medidas de latência podem não ser as mais indicadas para representar de forma precisa a resposta subcortical, sugerindo-se que medidas de frequência, em vez de temporais, poderiam ser um método mais adequado para mensurar a codificação neural em crianças com TF, conforme destacado em outro estudo^(44)^. Por isso, optou-se por ilustrar as diferenças na codificação neural entre os grupos avaliados através da análise do espectrograma (TFD) de um sujeito do GE e de um sujeito do GC (Figura 1), como exemplo dessa possibilidade de análise, que será discutida a seguir.

Ao se analisar a gravidade do TF e sua relação com os valores de latência dos componentes do FFR, observou-se que, quanto mais grave o TF, maior a latência, especialmente do componente E. Em conjunto, as ondas D, E e F são responsáveis pela codificação da estrutura sonora periódica e harmônica da vogal /a/^(42,43)^. Dessa forma, sugere-se que crianças com maior alteração de fala apresentam porção sustentada alterada, particularmente no componente E. Esse achado reforça a hipótese de que déficits na codificação neural dos sons da fala podem estar associados às dificuldades na produção articulatória. Como a porção sustentada do FFR está relacionada à estabilidade da representação neural de sons verbais, alterações neste parâmetro podem indicar um processamento auditivo subjacente comprometido.

Além disso, déficits na percepção e na manutenção da estrutura sonora das vogais podem impactar diretamente na aquisição fonológica e na inteligibilidade da fala, contribuindo para um padrão atípico de desenvolvimento da linguagem oral. Esses resultados destacam a importância de avaliações neurofisiológicas como o FFR na identificação precoce de alterações auditivas subjacentes aos TSF, como já foi evidenciado na literatura^(8,9)^. Acredita-se que em um futuro próximo, a compreensão dos padrões de latência e suas implicações no processamento da fala pode orientar abordagens terapêuticas mais eficazes, favorecendo estratégias de intervenção que otimizem a percepção e a produção dos sons da fala em crianças com TF. Ressalta-se, contudo, que a hipótese inicial era de que a gravidade do TF estaria diretamente proporcional ao aumento da latência de todos os componentes mensurados no FFR.

No que concerne à amplitude dos componentes do FFR e ao valor do *slope,* que reflete a relação entre o tempo (ms) e a magnitude (µV) da resposta neural do complexo VA^(43)^, presente estudo não identificou diferenças estatísticas significativas entre os grupos, contrariando as hipóteses inicialmente propostas. Analisando as possíveis explicações para esses resultados, especialmente ao compará-los com os de um estudo^(8)^ que investigou crianças com TF e observou diferenças nas latências de outros componentes do FFR (V, A, F, O), bem como no *slope*, sugere-se que a discrepância pode ser atribuída ao GC. Durante a coleta de dados deste estudo, realizada em 2023, no período pós-pandêmico, enfrentou-se dificuldades significativas para encontrar crianças sem alterações nas habilidades auditivas comportamentais, conforme mencionado anteriormente. Portanto, não se consideraram os resultados normais nos dois testes de PAC para os critérios de elegibilidade, dado que o foco principal era investigar as habilidades auditivas de integração binaural e de resolução temporal, além da codificação neural em crianças com TF, independentemente do *status* do PAC das crianças com fala típica.

Na análise simplificada do espectrograma, ao analisar visualmente cada uma dos registros dos diferentes sujeitos amostrais, notou-se diferenças na excitação neuronal entre os grupos estudados. A predominância da cor amarela, indicando maior ativação na faixa de 0-200Hz no sujeito do GC, sugere que essas manifestações podem estar relacionadas a alterações comportamentais significativas, detectadas por meio da eletrofisiologia, indicando uma menor ativação neuronal no GE, composto por crianças com TF. Essa faixa de frequência está diretamente associada ao tom vocal humano e à atividade de sincronização de fase (*phaselocking*)^(45)^. Além disso, diversos pesquisadores destacam a relevância do FFR na compreensão da codificação das informações sonoras, tanto a nível subcortical quanto cortical, apontando para a diferenciação nas respostas conforme os centros neurais envolvidos em cada patologia^(46)^.

Ressalta-se que a análise do espectrograma foi realizada apenas para fins didáticos e para melhor visualização de ativações neurofisiológicas, visto que, na elaboração da Figura 1, considerou-se apenas um sujeito amostral de cada grupo, escolhido de forma aleatória. Sugere-se que não sejam feitas generalizações e/ou demais inferências considerando tal comparação. Entretanto, este dado sinaliza mais um caminho potencial de pesquisas com o FFR em crianças com TSF, especialmente nas crianças com TF.

Este estudo reforça, mais uma vez, a importância da audição para a produção adequada da fala, justificando a inclusão de atividades de estimulação das habilidades auditivas no contexto terapêutico para crianças com TF, contribuindo para uma abordagem terapêutica mais ampla, direcionada e eficaz. Essa prática clínica é promissora, podendo até reduzir o tempo de intervenção fonoaudiológica, o que representa uma vantagem tanto para os serviços privados quanto, em especial, para os serviços públicos, sobrecarregados pela alta demanda de atendimento às crianças com TSF.

A principal limitação deste estudo pode residir na composição amostral do GC. Futuras investigações sobre outras habilidades auditivas seriam relevantes para uma compreensão mais abrangente de como o SNAC dessas crianças se comporta. Por fim, destaca-se a importância de uma análise frequencial mais detalhada da codificação neural da fala em crianças com TSF.

## CONCLUSÃO

Crianças com TF apresentam alterações na habilidade auditiva de integração binaural e na codificação neural dos sons da fala. Logo, tanto os resultados comportamentais quanto os eletrofisiológicos apontam que há possível relação entre a percepção auditiva e a produção de fala nas crianças com TF.
